# Functions of Conserved Domains of Human Polynucleotide Phosphorylase on RNA Oxidation

**DOI:** 10.36959/584/448

**Published:** 2019-09-22

**Authors:** Sulochan Malla, Zhongwei Li

**Affiliations:** Department of Biomedical Science, Florida Atlantic University, USA

**Keywords:** RNA oxidation, hPNPase, Catalytic domains, RNA binding domains

## Abstract

Human polynucleotide phosphorylase (hPNPase), an exoribonuclease that is primarily localized in mitochondria, plays an important role in reducing oxidized RNA and protecting cells under oxidative stress conditions. hPNPase contains two catalytic domains (RPH1 and RPH2) and two RNA binding domains (KH and S1), and an N-terminal mitochondrial translocation signal (MTS). In this study, we examined the potential roles of each domain in hPNPase function on controlling RNA oxidative damage. DNA encoding full-length hPNPase and its domain-deletion mutants were introduced into HeLa cells, and the levels of an oxidized RNA lesion, 8-hydroxyguanosine (8-oxo-Guo) were determined in mitochondrial and cytoplasmic RNA under oxidative stress conditions. Our study showed that the S1 RNA binding domain is crucial for reducing 8-oxo-Guo in both cytoplasm and mitochondria, while the MTS is required for 8-oxo-Guo reduction in mitochondria.

## Introduction

RNA oxidation has become increasingly recognized problem because it is strongly implicated in the pathogenesis of numerous human diseases, mostly neurodegeneration [1–5]. Recent studies suggest that oxidized RNA is deleterious, and organisms may have developed specific mechanisms by which oxidized RNA molecules are eliminated within cells before they exert detrimental effect [3,6]. One of such mechanisms may involve polynucleotide phosphorylase (PNPase) enzymes in both Escherichia coli and human cells since they showed high binding affinity to synthetic oligonucleotide containing oxidized lesion 8-hydroxygunosine (8-oxo-Guo) than normal RNA [7,8]. Later, it was reported that both *E. coli* and human PNPase are responsible for reducing 8-oxo-Guo in cellular RNA and for protecting cells against oxidative stress challenge [9].

PNPase is an evolutionarily conserved 3’ → 5’ exoribonuclease that participates in RNA metabolism and regulates diverse physiological processes including cellular senescence and homeostasis [10–15]. The human PNPase (hPNPase) contains two RPH catalytic domains and an α-helix in between them occupying the N-terminal two-third of the protein, and KH and S1 RNA binding domains (KH and S1) at the C-terminal. In addition, a mitochondrial-targeting signal (MTS) is present at its N-terminal region which helps to translocate hPNPase to mitochondria [13,15–18]. The finding that hPNPase is primarily localized in mitochondria is consistent with its function in turnover of mitochondrial mRNA as well as RNA transport into mitochondria [14,16,19–21]. Inasmuch as mitochondria is thought to be the primary generator of reactive oxygen species (ROS) in eukaryotic cells under physiological conditions, it is likely that hPNPase plays a role in reducing oxidation of mtRNA. Furthermore, hPNPase has also been reported to localize inside cytoplasm [13,22] where it may help reduce oxidized RNA in cytoplasm.

hPNPase may reduce the levels of oxidized RNA through specific binding, sequestration and subsequent degradation of RNA containing oxidized lesions [3]. These activities may be mediated separately by the enzyme’s specific catalytic and RNA binding domains. In this study, we examined the roles of these domains of hPNPase in regulating 8-oxo-Guo levels in cytoplasmic and mitochondrial RNA.

## Materials and Methods

### Chemicals and reagents

Diethyl pyrocarbonate (DEPC), tert-butyl hydroperoxide (tBHP), proteinase inhibitor cocktail, and chelex-100 were purchased from Sigma-Aldrich (St. Louis, MO). Tri Reagent-LS was obtained from Molecular Research Center, Inc. (Cincinnati, OH) and 8- hydrooxyguanosine (8-oxo-Guo) was purchased from Calbiochem (La Jolla, CA). Antibodies specific for hPNPase and hGAPDH were purchased from Abnova (Abnova Taipei, Taiwan). Lipofectamine 3000 Reagent was purchased from Life Technologies (Invitrogen, CA). Dulbecco’s Modified Eagle’s Media, growth bovine serum and penicillin/streptomycin solution were all purchased from Hyclone (Hyclone, CA).

### Cell culture and treatment

HeLa cells and culture conditions were previously described [9]. All cultures were maintained in Dulbecco’s Modified Eagle’s Media (DMEM) supplemented with 10% (v/v) heat inactivated growth bovine serum (GBS) and 1% penicillin/streptomycin solution at 37 °C under a humidified condition containing 5% CO_2_. Cells were grown up to 70–80% confluency at the time of transfection or treatment with the oxidants.

Construction of hPNPase domain-deletion mutants DNA constructs encoding the full length hPNPase and its domain-deletion mutants were synthesized, sequenced, and inserted downstream of a CMV promoter in the pcDNA3.1 expression vector (Genscript, Piscataway, NJ). The amino acid sequences that were deleted in the mutant proteins were made in the same as reported previously [13]. The resulting plasmids, pcDNA3.1-pnp, pcDNA3.1-ΔMTS, pcDNA3.1-ΔRPH1, pcDNA3.1-ΔRPH2, pcDNA3.1-ΔKH, and pcDNA3.1-ΔS1, were used to express the encoded proteins in this study. For convenience, the DNA will be referred as PNP, ΔMTS, ΔRPH1, ΔRPH2, ΔKH and ΔS1 in the subsequent discussions.

### Preparation of plasmid DNA

High efficiency NEB® 5-alpha competent *E. coli* cells were purchased from (New England Biolabs, Boston, MA) and the plasmids containing PNP and its mutants were transformed as per manufacturer’s instructions. Plasmid DNA were prepared from purified transformants using PureLink™ HiPure Plasmid Midiprep Kit (Thermo Fisher Scientific, Grand Island, NY) by following manufacturer’s instructions.

### Expression of cloned hPNPase

For the expression analysis of hPNPase and its mutants, cultured HeLa cells were transfected with PNP or domain deletion constructs using the Invitrogen’s Lipofectamine 3000 reagent (Thermo Fisher Scientific, Grand Island, NY) by following manufacturer’s protocol. Briefly, HeLa cells were cultured up to 70–80% confluency at the time of transfection. Lipofectamine reagent and plasmid DNA were diluted in serum-free Opti-MEM medium. Diluted Lipofectamine and plasmid DNA were mixed (1: 1) followed by incubation for 15 minutes at room temperature. The mixtures were added to the cell cultures and harvested after 72 hours for further analysis.

### Separation of mitochondrial and cytoplasmic fractions

Mitochondrial and cytoplasmic fractions were separated by following a procedure with slight modifications. Teflon coated glass homogenizer was used to disrupt the cells in the presence of TKMg buffer (10 mM Tris-HCL with pH 7.0, 10 mM KCL, 0.15 mM MgSO_4_ and freshly prepared DFOM at 1 mM). The homogenate was brought to a final concentration of 0.25 M sucrose, and was centrifuged at 1,500 g for 3 minutes. The supernatant was then centrifuged at 10,000 g for 10 minutes. Both pellet (crude mitochondrial fraction) and supernatant (cytoplasmic fraction) of the high-speed centrifugation were preserved. The mitochondrial fraction was further washed with TKMg buffer and centrifuged at 10,000 g for 10 minutes to remove contaminating cytoplasmic materials in the mitochondrial pellet. Finally, the pellet was suspended in freshly-prepared TKMg sucrose solution.

### RNA isolation

RNA was purified from mitochondrial and cytoplasmic fractions of HeLa homogenates using TRI Reagent-LS (Molecular Research Center, Inc., Boston, MA). The reagent was supplemented with freshly-prepared DFOM at a final concentration of 1 mM to prevent artificial RNA oxidation. Purified RNA was dissolved in RNase-free water, and was quantified using a ND 800 spectrophotometer.

### Quantification of 8-oxo-Guo by reverse-phase HPLC

RNA samples were digested into nucleosides using nuclease P1 and the resulting nucleosides were separated by HPLC and detected by UV and ECD detectors as described previously. The number of 8-oxo-Guo and guanine (G) in the RNA sample were calculated according to the retention time of standard 8-oxo-Guo and G.

### Statistical analysis

All experiments were carried out at least in triplicates. All data were presented as means ± SD of absolute values or percentage of control. Values were analyzed for statistical significance by Student’s t-test. P < 0.05 was considered significant.

## Results

### Expression of exogenous hPNPase reduces 8-oxo-Guo levels in both cytoplasm and mitochondria

**Figure 1:**
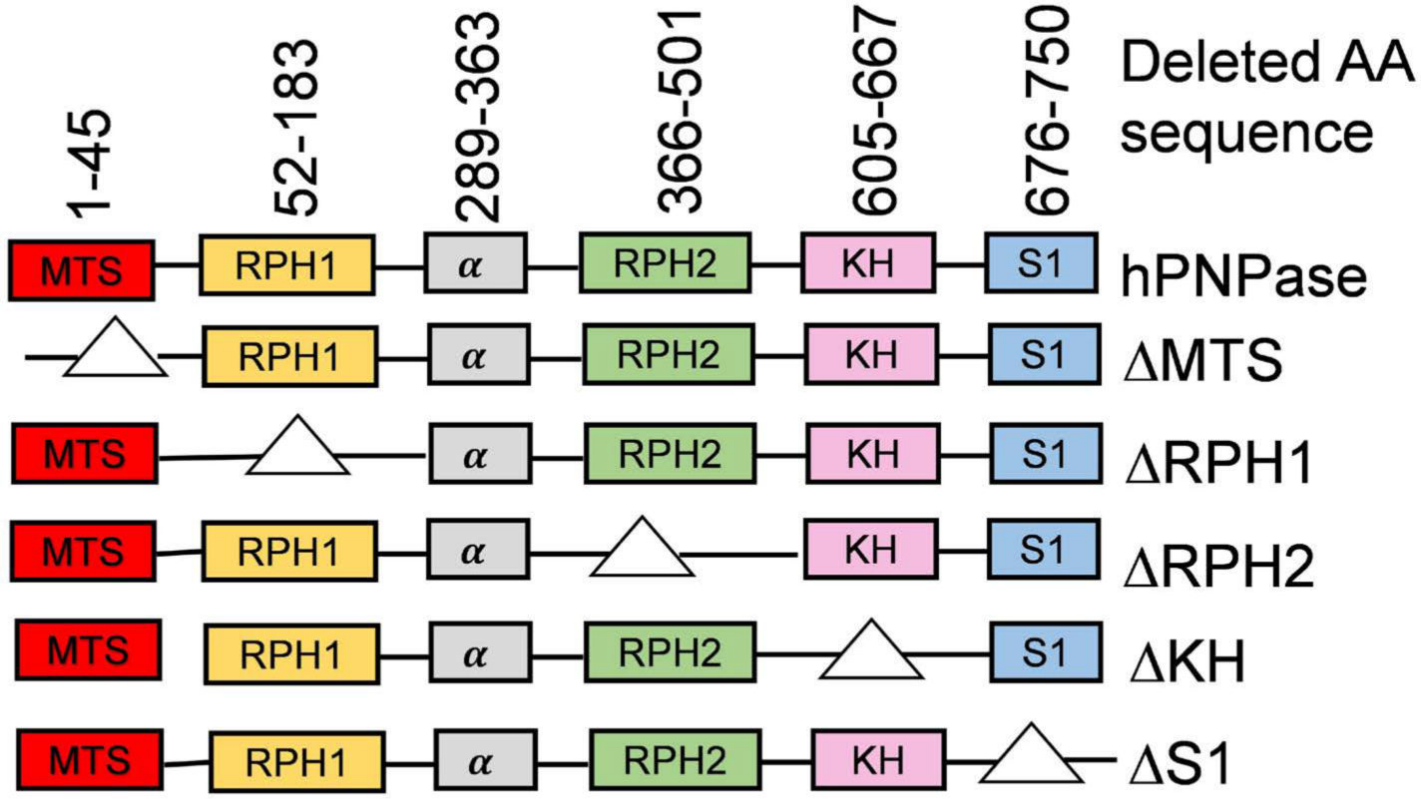
Diagrammatic representation of full-length hPNPase and its domain-deletion mutants. The amino acid (AA) sequences deleted in each of the domain-deletion mutants are the same as previously described [13].

In order to examine the roles of individual domains of hPNPase in regulating RNA oxidation levels, we have constructed full-length PNPT1 gene which can be expressed under the control of a CMV promoter in the vector pcDNA3.1. Mutants missing one of the domains were also constructed in the same expression system. The amino acid sequences that were deleted in each of these mutant proteins is described in Figure 1. Upon transfection of the PNP construct, HeLa expresses the encoded proteins at levels 2–3 folds of the endogenous hPNPase based on western blotting (data not shown). However, expression of most mutants could not be shown, presumably due to failure of binding by the antibody. The same phenomenon was observed in a previous report when the same mutant proteins were subjected to western blot analysis [18]. However, based on that work, and the results below, the proteins were made and their function, if any, can be detected.

**Figure 2:**
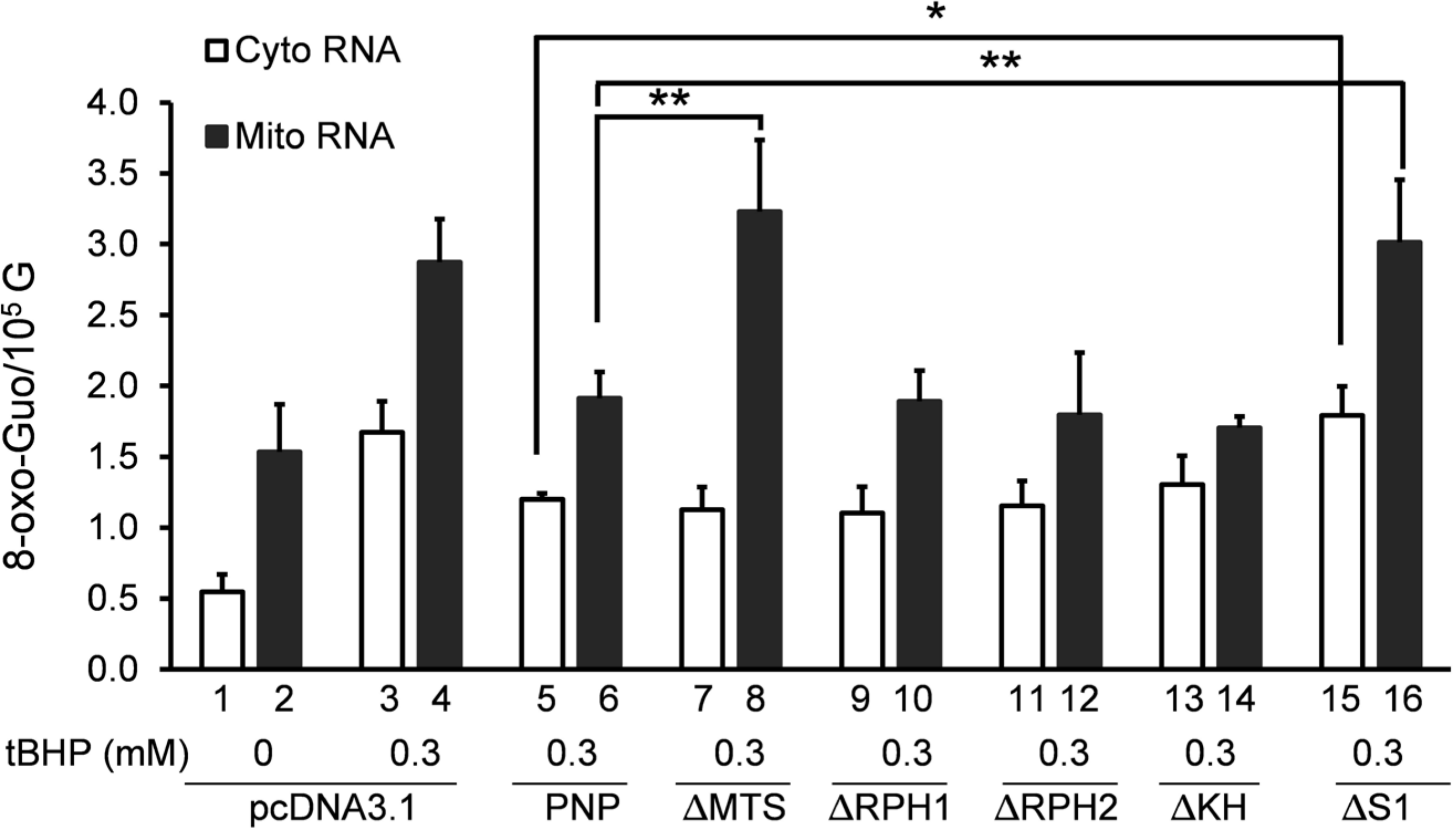
The 8-oxo-Guo levels in mitochondrial and cytoplasmic RNA in HeLa cultures transfected with DNA constructs encoding hPNPase and its mutants. The constructs that were introduced into HeLa are labeled on the bottom of the bars, indicating the plasmid vector (pcDNA3.1), DNA encoding the full-length hPNPase (PNP) and domain-deletion mutants (ΔMTS, ΔRPH1, ΔRPH2, ΔKH and ΔS1) deletion mutants. Cultures treated with tBHP for 3 hours at 0 or 0.3 mM are indicated above the names of the constructs. The data represent the means ± standard deviations of three independent experiments with significance levels at *: P < 0.05 and **: P < 0.01, between the marked pairs of bars.

We first investigated the role of full-length hPNPase on 8-oxo-Guo levels in mitochondria and cytoplasm. In cultures transfected with the vector (pcDNA3.1), 8-oxo-Guo level in mitochondrial RNA is about 1.5 8-oxo-Guo per 105 G, nearly 3 times higher than in cytoplasmic RNA (Figure 2, Bar 1 and Bar 2). This is also true in cells without transfecting the vector (data not shown). The 8-oxo-Guo levels in both mitochondria and cytoplasm increased significantly when cells were treated with 0.3 mM of tBHP for 3 hours, going up almost 3 times in cytoplasm and 2 times in mitochondria, respectively (Figure 2, Bar 3 and Bar 4).

As anticipated, introduction of DNA encoding the full-length hPNPase (PNP) caused a decrease of the level of tBHP-induced 8-oxo-Guo in both mitochondrial and cytoplasmic RNA in response to 0.3 mM tBHP treatment (Figure 2, Bar 5 and Bar 6). While the level of 8-oxo-Guo in cytoplasmic RNA (Figure 2, Bar 5) is reduced by nearly 30% compared to that with vector DNA (Figure 2, Bar 3), slightly more reduction in 8-oxo-Guo level was found in mitochondrial RNA when compared to that with vector DNA (Figure 2, compare Bar 6 with Bar 4). This result is consistent with our earlier finding that hPNPase reduces the level of 8-oxo-Guo in total RNA in HeLa cells when it is overexpressed, and vice versa [9].

As described below, results of 8-oxo-Guo levels following introduction of DNA encoding a domain-deletion mutant will be compared to those resulted from introduction of the vector or the PNP DNA encoding full-length hPNPase. Introduction of PNP caused a reduction of 8-oxo-Guo in both cytoplasmic and mitochondrial RNA. If a mutant loses the ability to reduce 8-oxo-Guo in RNA of any fraction, it would suggest a role of the specific domain in eliminating oxidized RNA in that cellular fraction.

### MTS is required to reduce tBHP-induced 8-oxo-Guo in mitochondrial RNA

As described previously, higher organisms such as mammals and plants have an N-terminal peptide sequence that is responsible for the enzyme’s translocation to mitochondria and chloroplast, respectively [16,23]. We were interested in exploring the effect of MTS-deleted hPNPase on 8-oxo-Guo levels of mitochondrial and cytoplasmic RNA. The rationale of this particular study is that hPNPase will not be able to translocate to mitochondria upon deletion of its MTS domain and it may not be able to function inside mitochondria. Consequently, the level of mitochondrial 8-oxo-Guo may not be effectively reduced by introduction of the ΔMTS construct, compared to the introduction of wild type hPNPase. Towards this goal, fully conserved amino acid residues of MTS domain (1–45) were chosen as targets for mutagenesis as shown in Figure 1. The resulting construct ΔMTS was transfected to cultured HeLa cells and its effect on 8-oxo-Guo levels in RNA was compared to those transfected with the PNP DNA encoding full-length hPNPase to reveal the specific role of MTS.

Upon introduction of ΔMTS, tBHP-induced 8-oxo-Guo level in cytoplasmic RNA (Figure 2, Bar 3) is reduced to the same level as it did following the introduction of PNP (Figure 2, Bar 5). This indicates that the ΔMTS protein was expressed and functional to a similar level as the PNP protein. Remarkably, introduction of ΔMTS showed a higher tBHP- induced 8-oxo-Guo level in mitochondrial RNA (Figure 2, Bar 8) compared to those transfected with PNP DNA (Figure 2, Bar 6). After introduction of ΔMTS, the level of 8-oxo-Guo in mitochondrial RNA is almost the same as the introduction of vector DNA (Figure 2, Bar 4). This observation suggests that exogenously expressed hPNPase may not be able to translocate to the mitochondria when its MTS domain is deleted. The result presents further evidence that hPNPase is a significant player of oxidized RNA control mechanism.

### Either of the RPH1 or RPH2 domains is sufficient to reduce 8-oxo-Guo levels in RNA under oxidative stress conditions

Next, we examined the potential roles of RPH1 and RPH2 catalytic domains in reducing 8-oxo-Guo levels in RNA. For this purpose, the ΔRPH1 and the ΔRPH2 constructs were generated by deleting the amino acids residues of RPH1 (52–183) or RPH2 (366–501) domains as described in Materials and Methods (Figure 1). Surprisingly, introduction of either construct caused reductions in tBHP-induced 8-oxo-Guo levels (Figure 2, Bar 9, Bar 10, Bar 11 and Bar 12) that are similar to those caused by PNP DNA (Figure 2, Bar 5 and Bar 6). Consistent with the finding of a previous report using domain-deletion constructs of the same nature [13], the results shown here indicate that either of the RPH domains may support the activities of full-length hPNPase to reduce the level of oxidized RNA during oxidative stress condition.

### S1 but not KH RNA-binding domain is responsible for reducing tBHP-induced 8-oxo-Guo levels

hPNPase binds to oxidized RNA with high affinity [8]. This important activity of hPNPase may be related to its function for reducing oxidized RNA in HeLa cells [9]. It is likely that hPNPase’s high binding affinity to oxidized RNA is attributed to its conserved RNA binding domains. As described previously, hPNPase has two RNA binding domains i.e. KH (605–667) and S1 (676–750) at the C-terminal end [13,15,17]. These RNA binding domains play an important role in various biological processes [13]. To examine the role of KH and S1 binding domains in oxidized RNA control mechanism, DNA constructs encoding hPNPase lacking KH or S1 domains, i.e. ΔKH and ΔS1, were introduced to HeLa cell cultures, and the levels of tBHP-induced 8-oxo-Guo in mitochondrial and cytoplasmic RNA were examined.

Upon introduction of ΔKH, the levels of 8-oxo-Guo in mitochondrial and cytoplasmic RNA (Figure 2, Bar 13 and Bar 14) were essentially the same as those with introduction of PNP DNA encoding the full-length hPNPase (Figure 2, Bar 5 and Bar 6) in response to tBHP treatment. This suggested that KH domain is indispensable for reducing 8-oxo-Guo levels in either cytoplasmic or mitochondrial RNA. In contrast, introduction of the ΔS1 DNA caused a complete loss in the ability to reduce 8-oxo-Guo in both cytoplasmic and mitochondrial RNA (Figure 2, Bar 15 and Bar 16). This result indicates that S1, but not KH, is essential for hPNPase to reduce 8-oxo-Guo levels in RNA. Whether S1 domain directly contributes to the binding of oxidized RNA at high affinity requires further analysis in the future.

## Discussion

In this study, we have examined the roles of various functional domains of hPNPase in controlling the levels of an RNA oxidation marker (8-oxo-Guo) in both cytoplasmic and mitochondrial fractions. In particular, hPNPase mutants lacking specific functional domains, i.e., mitochondrial translocation signal (MTS), catalytic domains (RPH1 and RPH2) and RNA binding domains (KH and S1), were exogenously expressed in cultured HeLa cells. The effects of domain-deletion on the levels of 8-oxo-Guo were analyzed in comparison with full-length hPNPase. Our data showed that: 1) MTS is required for hPNPase to reduce 8-oxo-Guo in mitochondria, but not in cytoplasm; 2) either RPH1 or RPH2 domain alone is able to support the full activity of hPNPase in reducing 8-oxo-Guo during oxidative stress, and 3) the S1 RNA-binding domain, but not KH, is required for hPNPase to reduce 8-oxo-Guo under oxidative stress. Taken together, these results imply that hPNPase controls oxidized RNA in both cytoplasm and mitochondria, presumably by binding and degrading oxidized RNA depending on its individual functional domains.

Data presented here suggested that hPNPase may play a multifunctional role in controlling RNA oxidation during oxidative stress condition. We have previously proposed that various factors may bind to and sequester oxidized RNA, and eventually eliminate the RNA within cells, thus preventing deleterious effect of oxidized RNA [3]. Our present findings are consistent with the idea that hPNPase binds oxidized RNA specifically, and participate in the degradation of the RNA.

The S1 domain is likely important for binding of oxidized RNA. Deletion of S1 completely abolished the activity of hPNPase in reducing cytoplasmic and mitochondrial 8-oxo-Guo. This result suggests that S1 domain may first bind to oxidized RNA before the catalytic domains could participate in degradation. Conversely, when this binding domain is missing then hPNPase may not bind to oxidized RNA and downstream activities such as degradation and elimination could be impeded. Interestingly, the KH binding domain has no role in reducing oxidized RNA as evidenced by the unaltered 8-oxo-Guo level when this particular domain is obliterated. This is contrary to the earlier findings where the integrity of KH and S1 domains in *E. coli* PNPase are instrumental in normal RNA binding, degradation and polyadenylation activities [24]. Moreover, the crystal structure analysis of the S1 and KH domains of hPNPase revealed a conserved GXXG motif in KH has an active role in binding to normal RNA [25]. They also reported that KH pore traps the long RNA 3’ tail to deliver them to RPH channel for further degradation. Our results suggest that the selected binding of hPNPase to oxidized RNA can be different from the binding of normal RNA involving different combinations of RNA-binding domains.

Deletion of either RPH1 or RPH2 domain do not seem to affect the activity of hPNPase in dealing with oxidized RNA as the levels of mitochondrial and cytoplasmic 8-oxo-Guo were similar when expressing PNP, ΔRPH1 or ΔRPH2 (Figure 2). This finding insinuates that the presence of any one of these domains are enough for hPNPase to eliminate 8-oxo-Guo in oxidized RNA. Remarkably, our findings closely resemble previous observations showing that the presence of at least one RPH1 domain can retain functionality of hPNPase [13]. Interestingly, they also showed that RNA degradation activity was still preserved when both RNA binding domains (KH and S1) were deleted suggesting that RPH domains may also bind themselves to RNA [13]. This behavior is contrary to what was known for the RPH domains of bacterial and chloroplast PNPase in degradation of normal RNA. It was shown that mutation in the key residues of any one of these catalytic domains inhibited PNPase activity in bacteria [26]. Similarly, chloroplast RPH1 domains are highly active enzymatically but RPH2 exhibited low RNA degradation activity of non-polyadenylated RNA [23]. Future studies should be directed toward understanding the role of hPNPase in controlling oxidized RNA when both catalytic domains and RNA binding domains are inactivated.

The activities of hPNPase for selected elimination of oxidized RNA in cytoplasm and mitochondria rely on its localization in both cellular compartments which depends on MTS and may be regulated under particular conditions. The localization of hPNPase has been debated among various groups. Some reported that hPNPase is a mitochondrial intermembrane protein whereas other claimed that it is predominantly localized in the mitochondrial matrix [20,21,27]. More recently, it is shown that hPNPase forms a complex with various other proteins, including RNA helicase hSUV3, GSRF protein, and mitochondrial poly (A) polymerase in distinct foci in mitochondria matrix in the form of degradosome. These complexes are responsible for various activities such as RNA processing, degradation, and import into mitochondria [20]. Our present work suggest that MTS is required for hPNPase function in reducing 8-oxo-Guo in mitochondria, but it is dispensable in cytoplasm. This is consistent with the expected function of MTS for the protein’s predominant localization in mitochondria. However, hPNPase must be present in cytoplasm since its function in degrading cytoplasmic RNA has been repeatedly identified [13,22]. In concert with this idea, a reduction in the cytoplasmic 8-oxo-Guo as was observed in this work (Figure 2) when PNP or ΔMTS were introduced. It remains to be examined whether endogenous hPNPase translocates between cytoplasm and mitochondria, especially in response to certain conditions such as oxidative stress.

## Abbreviations

8-oxo-Guo: 8-hydroguanosine
DEPC: Diethyl Pyrocarbonate
DFOM: Deferoxamine Mesylate
hPNPase: Human Polynucleotide Phosphorylase
hSUV3: Human RNA Helicase
MTS: Mitochondrial Translocation Signal
RPH1: RNase PH Domain 1
RPH2: RNase PH Domain 1
KH: K-Homology
tBHP: tertiary Butyl Hydroperoxide
